# Truly a hyperparasite, or simply an epibiont on a parasite? The case of *Cyclocotyla bellones* (Monogenea, Diclidophoridae)

**DOI:** 10.1051/parasite/2022028

**Published:** 2022-05-19

**Authors:** Chahinez Bouguerche, Fadila Tazerouti, Jean-Lou Justine

**Affiliations:** 1 Department of Zoology, Swedish Museum of Natural History Box 50007 104 05 Stockholm Sweden; 2 Université des Sciences et de la Technologie Houari Boumediene, Faculté des Sciences Biologiques, Laboratoire de Biodiversité et Environnement: Interactions - Génomes BP 32 El Alia Bab Ezzouar, Alger Algérie; 3 Institut Systématique Évolution Biodiversité (ISYEB), Muséum National d’Histoire Naturelle, CNRS, Sorbonne Université, EPHE, Université des Antilles 57 rue Cuvier CP 51 75231 Paris Cedex 05 France

**Keywords:** hyperparasitism, Epibiosis, *Cyclocotyla bellones*, Cymothoidae, Nutrition, Adaptation

## Abstract

*Cyclocotyla bellones* Otto, 1823 (Monogenea, Diclidophoridae) is one of the few monogenean species reported as hyperparasitic: the worms dwell on cymothoid isopods, themselves parasites of the buccal cavity of fishes. We present here observations based on newly collected monogenean specimens from *Ceratothoa parallela* (Otto, 1828), an isopod parasite of *Boops boops* off Algeria and also investigated its diet to address whether *Cy. bellones* is indeed a hyperparasite, i.e., whether it feeds on the isopod. We also compared the body shape of various monogeneans belonging to the same family as *Cy. bellones*, the Diclidophoridae, including *Choricotyle* cf. *chrysophryi* Van Beneden & Hesse, 1863, collected from *Pagellus acarne* off Algeria. No morphological character of the anterior organs suggested any special adaptation in *Cy. bellones* to the perforation of the crustacean cuticle. The wall of the oesophagus and of the intestine of *Cy. bellones* was lined with a dark pigment similar to what is usually observed in haematophagous polyopisthocotyleans, and which is derived from ingested fish blood. We noticed that an anterior elongate stem exists only in diclidophorids dwelling on parasitic isopods and never in those attached to the gills. We hypothesize that the anterior stem of the body of *Cy. bellones* is an anatomical adaptation for the monogenean to feed on the fish while dwelling on the isopod. We thus consider that *Cy. bellones* is an epibiont of the parasitic crustacean, as it uses it merely as an attachment substrate, and is not a true hyperparasite.

## Abstract (fr)

*Cyclocotyla bellones* Otto, 1823 (Monogenea, Diclidophoridae) est l’une des rares espèces de monogènes signalées comme hyperparasites : les vers vivent sur des isopodes cymothoïdes, eux-mêmes parasites de la cavité buccale des poissons. Nous présentons ici des observations basées sur des spécimens de monogènes nouvellement collectés de *Ceratothoa parallela* (Otto, 1828), un isopode parasite de *Boops boops* au large de l’Algérie et avons également étudié son régime alimentaire pour déterminer si *Cy. bellones* est bien un hyperparasite (c’est-à-dire, se nourrit-il de l’isopode ?). Nous avons également comparé la morphologie de divers monogènes appartenant à la même famille que *Cy. bellones*, les Diclidophoridae, dont *Choricotyle* cf. *chrysophryi* Van Beneden & Hesse, 1863, collecté sur *Pagellus acarne* au large de l’Algérie. Aucun caractère morphologique des organes antérieurs ne suggérait d’adaptation particulière à la perforation de la cuticule des crustacés chez *Cy. bellones*. La paroi de l’œsophage et de l’intestin de *Cy. bellones* était tapissée d’un pigment foncé semblable à ce que l’on observe habituellement chez les Polyopisthocotylea hématophages, et qui est issu du sang de poisson ingéré. Nous avons remarqué qu’une partie allongée antérieure n’existe que chez les Diclidophoridae vivant sur des isopodes parasites et jamais chez ceux attachés aux branchies. Nous émettons l’hypothèse que la partie antérieure du corps de *Cy. bellones* est une adaptation anatomique permettant au monogène de se nourrir du poisson tout en vivant sur l’isopode. Nous considérons donc que *Cy. bellones* est un épibionte du crustacé parasite, puisqu’il ne l’utilise que comme substrat pour son attachement, et n’est pas un véritable hyperparasite.

## Introduction

Parasitism is a bipartite association in which the parasite depends on the host, deriving benefits [85] such as food, habitat and locomotion [29]. A tripartite interaction, or hyperparasitism, is found when a parasite is parasitic on another parasite [44]; this is considered a highly evolved mode of living [44]. A quadripartite interaction, or hyper-hyperparasitism also exists, but we are aware of a single case described on fish: the flagellate *Cryptobia udonellae* Frolov & Kornakova, 2001 on the monogenean *Udonella murmanica* Kornakova & Timofeeva, 1981, itself on the copepod *Caligus curtus* Müller, 1785, finally itself on the fish *Gadus morhua* Linnaeus [42].

Dollfus published in 1946 an astounding 483-page compilation of all cases known to him of hyperparasites of helminths [35]. We list here a few recent studies, limited to parasites of fish helminths. Nematodes can parasitize nematodes [68] and cestodes [83]. Microsporidians and myxozoans can also parasitize digeneans [17, 57, 66, 73, 93], monogeneans [1, 16], cestodes [87] and acanthocephalans [30, 62]. Dinoflagellates [27, 28] and bodonid flagellates [7] can parasitize monogeneans. Viruses have been reported from various fish helminths [48, 49] and many more may exist [32]; all these helminth viruses are thus hyperparasites. Hyperparasites were frequently reported from parasitic copepods, including microsporidians [41] and a leech [79]. However, Ohtsuka et al. (2018) recently reviewed all organisms living on parasitic copepods but considered that most of them were epibionts, not parasites [70].

Amongst fish-parasitic isopods, cymothoid isopods, including some species also known as “tongue replacers” [88], are frequently hosts of monopisthocotylean monogeneans, mainly udonellids (see [88]) and of polyopisthocotylean monogeneans, mainly diclidophorids (Table 1).

**Table 1:** Some hyperparasitic monogeneans on parasitic crustaceans.

Parasite	Type host	Type locality	References
*Cyclocotyla bellones* Otto, 1823	*Belone belone*	Italy, Mediterranean Sea	[72]
*Allodiclidophora charcoti* (Dollfus, 1922) Yamaguti 1963	*Ceratothoa oestroides* (female), buccal cavity of *Trachurus trachurus* and of *Boops boops*	Monaco, Mediterranean Sea; Spain, Atlantic Ocean	[33, 34]
*Choricotyle smaris* (Ijima, *in* Goto, 1894) Llewellyn, 1941	Cymothoa of buccal cavity of *Spicara smaris*	Italy, Mediterranean Sea	[45, 60]
*Allodiclidophora squillarum* (Parona & Perugia, 1889)	Ovigerous lamellae of *Bopyrus squillarum*	Italy, Mediterranean Sea	[76]
*Diclidophora merlangi* (Kuhn, *in* Nordmann, 1832)	Ceratothoa oestroides, buccal cavity of *Boops boops*	Italy, Mediterranean Sea	[33, 34]
*Choricotyle elongata* (Goto, 1894) Llewellyn, 1941	Mouth cavity of *Dentex tumifrons*, Cymothoa of mouth cavity*	Japan, Pacific Ocean	[45, 60]
*Choricotyle aspinachorda* Hargis, 1955	Gills and *Cymothoidae* from ventral pharyngeal region of *Orthopristis chrysopterus*	USA, Atlantic Ocean	[47]
*Udonella* spp.	For hosts and references, see Table 8.1. in [88]		
*Capsala biparasitica* (Goto, 1894)	Copepoda, probably of the genus *Parapelatus* sp. on gills of *Thynnus albacora*	Japan, Pacific Ocean	[45]

* The single specimen that was attached to the *Cymothoa* must be regarded as accidental [45].

**Table 2:** Diclidophorids from isopods, mouth and gills, used for body comparison. +, present. –, absent.

Species	Habitat	Anterior stem	Reference, page
*Cyclocotyla bellones*	*Belone belone*	+	[72] Plate XLI, Figs. 2.a–b
	Isopod of *Trachurus trachurus*	+	[34] p. 287, Fig. 1
	Isopod of *Boops boops*	+	[33] p. 349, Fig. 1; [36] p. 184, Fig. 2; [61] p. 136, Fig. 1.a; [84] p. 496 Plate 1; present study
	Isopod of *Spicara smaris*	+	[80] p. 102, Fig. 38; [9] p. 119, Fig II.VII
*Diclidophora merlangi* (Kuhn, *in* Nordmann, 1832)	Isopod of *Boops boops*	+	[96] Plate VII Fig. 1.b, Fig. 2
*Allodiclidophora squillarum* (Parona & Perugia, 1889)	*Bopyrus squillarum*	+	[76] p. 81, Fig. 1.a
*Choricotyle smaris* (Ijima, *in* Goto, 1894)	Isopod of *Spicara smaris*	+	[45] p. 208, wood-cut 1
*Neoheterobothrium affine* (Linton, 1898)	mouth of *Paralichthys dentatus*	+	[58] Plate XL Fig. 10
*Choricotyle elongata* (Goto, 1894)	Mouth cavity of *Dentex tumifrons*, *Cymothoa of mouth cavity*	–	[45] Plate X Fig. 9
*Orbocotyle prionoti* (MacCallum, 1917)	Gills of *Prionotus carolinus*	–	[63] Fig. 18
*Choricotyle* cf. *chrysophryi*	Gills of *Pagellus bogaraveo*	–	[60] p. 399, Fig. 2
Van Beneden & Hesse, 1863	*Pagellus acarne*		Present study
*Echinopelma neomaenis* (MacCallum, 1917)	Gills of *Lutjanus analis*	–	[63] Fig. 20
*Choricotyle labracis* (Cerfontaine, 1895)	Gills of *Dicentrarchus labrax*	–	[60] p. 421, Fig. 3
*Choricotyle hysteroncha* (Fujii, 1944)	Gills of *Haemulon striatum*, *Haemulon chrysargyreum*, *Haemulon flavolineatum*	–	[43] p. 157, Plate I Fig. 6
*Choricotyle multaetesticulae* (Chauhan, 1945)	Gills of *Pellona* sp.	–	[25] p. 137, Fig. 11
*Hargicotyle louisianensis* (Hargis, 1955)	Gills of *Menticirrhus americanus*	–	[47] p. 383, Fig. 8

Here, as part of an ongoing effort to characterise the parasite biodiversity of fishes off the Southern shores of the Mediterranean Sea [4–6, 12–15, 20–23, 31, 53–55, 69, 90], we present an illustrated redescription of *Cyclocotyla bellones* based on material from cymothoid isopods on *Boops boops* (Linnaeus). Previously, in a triple barcoding study, we confirmed the identity of the hyperparasite monogenean, the crustacean parasite-host and the primary fish hosts [15]. Here, we mainly address the question of whether the monogenean is truly a hyperparasite, i.e., does it feed on the isopod? We provide some information on the diet of the monogenean, compare its morphology to a close non-hyperparasite monogenean of the same family, and conclude that it probably feeds on the fish, not the isopod.

## Material and methods

### Collection and sampling of fish

Fish specimens were collected directly from local fishermen in Bouharoun (36°37′ N, 2°39′ E) and from fish markets in Réghaïa, Algerian coast, transferred to the laboratory shortly after purchase, identified using keys [38] and examined fresh on the day of purchase. Gill arches were removed and placed in separate Petri dishes. The buccal cavity, isopods and removed gills were observed under microscope for the presence of monogeneans.

### Collection of isopods and monogeneans

Monogeneans (*Choricotyle* cf. *chrysophryi*) parasitic on gills of the Axillary seabream *Pagellus acarne* (Risso) were simply removed from the gills. For the bogue, *Boops boops*, visibly infected isopods (those with visible monogeneans) were removed from the buccal cavity and sometimes photographed using a Leitz microscope. Monogeneans were removed from the isopods with a fine dissecting needle. Fish gill arches were carefully examined for the presence of monogeneans.

### Morphological methods

Monogeneans were fixed in 70% ethanol, stained with acetic carmine, dehydrated in an ethanol series (70, 96 and 100%), cleared in clove oil, and mounted in Canada balsam. Drawings were made with a Leitz microscope equipped with a drawing tube. Drawings were scanned and redrawn on a computer with Adobe Illustrator (CS5). Measurements are in micrometres, and indicated as means and between parentheses, the range. The nomenclature of clamp sclerites proposed by Llewellyn [60] and used by Euzet and Trilles [36] for *Cyclocotyla bellones* is adopted here.

To compare body shapes across diclidophorids from parasitic isopods infecting their hosts’ mouth and gills, figures in the global literature were extracted from published PDF files following [50]. The outlines of the body were drawn with Adobe Illustrator and then filled in black.

## Results

### Morphology of *Cyclocotyla bellones* Otto, 1823 (Figs. 1–3)

*Type-host*: *Belone belone* (Linnaeus), garfish (Belonidae) (but see Discussion).

**Figure 1:**
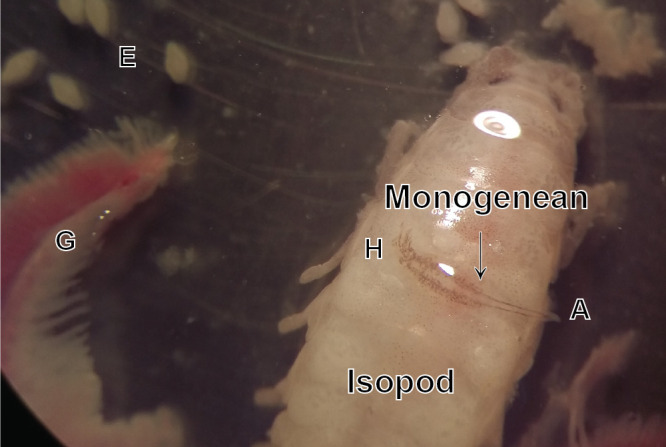
Photograph of *Cyclocotyla bellones*, on an isopod, *Ceratothoa parallela*, from the buccal cavity of the bogue *Boops boops*. E, egg. G, gill. H, haptor of the monogenean. A, anterior stem of the monogenean.

**Figure 2:**
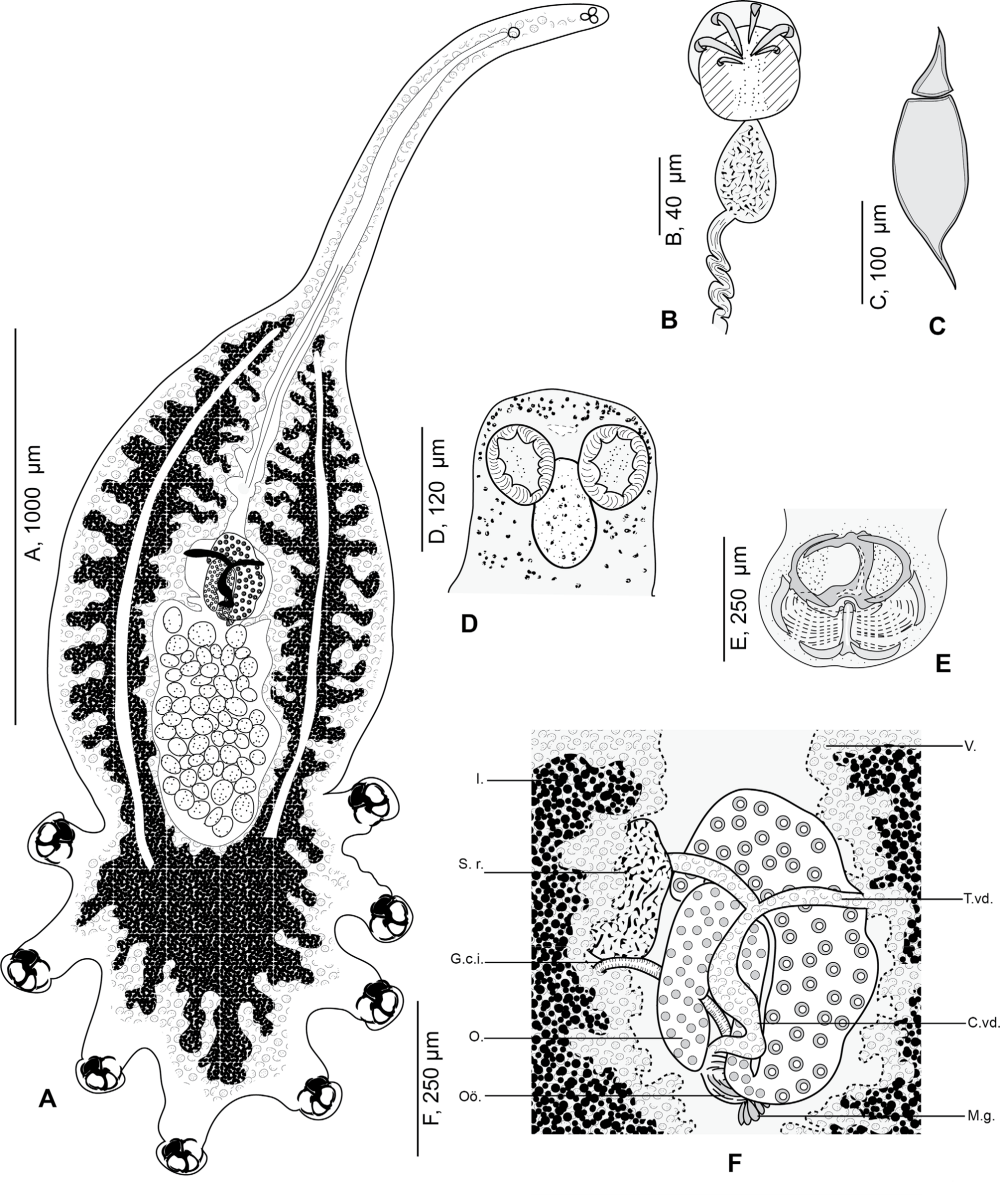
*Cyclocotyla bellones* Otto, 1823, specimen from *Ceratothoa parallela* from *Boops boops*, Algeria. A, whole body, MNHN HEL1312 (reproduced from Bouguerche et al., 2021 [15]); B, male copulatory organ, MNHN HEL1313; C, egg, MNHN HEL1314; D, anterior part, MNHN HEL1313; E, clamp, MNHN HEL1316; F, anatomy at level of ovarian zone, MNHN HEL1315. V., vitellarium. T.vd., transverse vitelloduct. C.vd., common vitelloduct. M.g., Mehlis’ glands. I., intestine. S.v., seminal vesicle. G.c.i., genito-intestinal canal. O., ovary. Oö., oötype.

**Figure 3:**
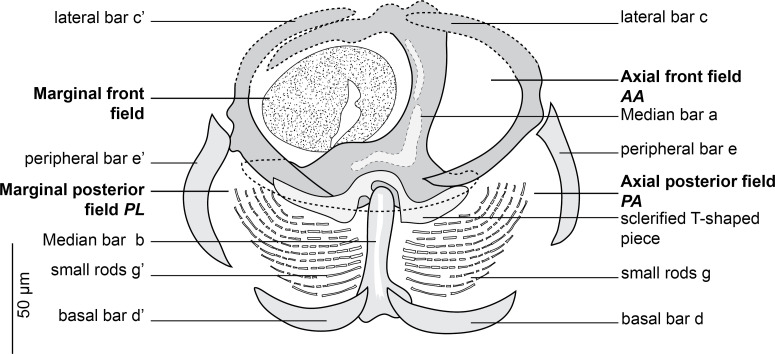
Clamp of *Cyclocotyla bellones* Otto, 1823, MNHN HEL1316. Nomenclature of the clamp sclerites as proposed by Llewellyn [60] and used by Euzet and Trilles [36].

*Additional hosts*: *Bopyrus squillarum* Latreille, 1802 (Bopyridae Rafinesque, 1815). Isopods of *Spicara maena* (Linnaeus) (Sparidae), the blotched picarel; of *Spicara smaris* (Linnaeus), the picarel; and of *B. boops* (Linnaeus) (Sparidae), the bogue. *Ceratothoa parallela* (Otto, 1828) from *B. boops* (this paper).

*Type-locality*: Italy [72].

*Additional localities*: Montenegro, France, and Turkey. Off Bouharoun, Algeria (36° 37′ 24″ N, 2° 39′ 17″ E) (this paper).

*Specimens from Algeria, from* Ceratothoa parallela *(Cymothoidae Leach, 1818) from the buccal cavity of* B. boops (Fig. 1). List of vouchers deposited in the collections of the Muséum National d’Histoire Naturelle, Paris, see [15].

Habitat: In the present study, *Cy. bellones* was most frequently observed on the upper part of the pereon of the isopod, occurring rarely on its pleon. A single specimen was found unattached in a Petri dish containing gills and an isopod, but there was no indication that the monogenean was detached from the gills rather than from the isopod.

Based on 14 specimens. Measurements in Table 3. Body elongate and fusiform, divided into three merged regions: a tapered anterior region; an enlarged middle region; and a posterior region formed by the haptor (Fig. 2A). Anterior region remarkably slender and attenuated, formed by an anterior lengthening, up to one-third of the total length in some specimens. No special feature found in mouth, prohaptor and anterior glands. Middle region rounded, containing reproductive system and extensive vitellarium follicles.

**Table 3:** Measurements of *Cyclocotyla bellones* from different hosts and localities.

Source	Dollfus, 1922 [33]	Dollfus, 1922 [34]	Euzet & Trilles, 1961 [36]	Lopez-Roman & Guevara Pozo 1976 [61]	Radujkovic & Euzet 1989 [80]	Present study
Host	*Ceratothoa oestroides* on *Trachurus trachurus*	*Ceratothoa oestroides* on *Boops boops*	Isopod on *Boops boops* and *Spicara maena*	*Ceratothoa oestroides* on *Boops boops*	Cymothoidae, buccal cavity of *Spicara smaris*	Cymothoidae, buccal cavity of *Boops boops*
Locality	Spain, Atlantic Ocean	Monaco, Mediterranean	France, Mediterranean	Alboran sea, Mediterranean	Montenegro, Mediterranean	Algeria, Mediterranean
Body length	3000		3000–8000	3600–7570	3000–8000	3900 (3150–6628)
Haptor length						1273 (557–17,729)
Anterior lobe length	650	2000		1800–3600		1970 (800–3207)
Total length						5046 (5100–8400)
Total width	2500		1000–4000	1580–1600	1000–4000	1536 (450–2300)
Clamps length	400*		200*			
Clamps width						
Buccal organ length			120*			42 (23–55)
Buccal organ width						44 (28–71)
Pharynx length			180			58 (38–74)
Pharynx width			110			51 (31–61)
Atrium length						44 (32–51)
Atrium width						46 (30–63)
Number of hooks		6**	6			6–8
Genital hooks length			60			17 (14–18)
Distance pharynx-anterior end						188 (101–308)
Distance genital atrium anterior end			500			383 (184–548)
Number of testes			40–90		40–90	

* Diameter.

** Number deduced from drawings. Note that all localities are from the Mediterranean Sea except Dollfus 1922, Atlantic.

Haptor semicircular, rounded to oval, bearing four pairs of pedunculated clamps (Fig. 2E). Peduncles cylindrical, long and thick, containing in anterior parts the intestinal diverticula and vitelline follicles; fourth pair of peduncles in a straight line to longitudinal body axis; first and fourth pairs of peduncles smaller.

Clamps circular (Fig. 3), typically diclidophorid in structure. Clamps with two regions, an anterior region and a posterior region delimited by 8 large bars in four fields: axial front field AA, marginal front field AL in anterior part; axial posterior field PA and marginal posterior field PL in posterior part. Anterior region hemispherical in flattened specimens, propped by 3 bars: *a*, *c* and *c’*. Median bar *a*, the largest. Dorsal arm of *a* ending in a long elbow on proximal side and in a T with unequal branches on distal side. A sclerotised T-shaped piece hinging ventrally to *a*. Ventral arm of *a* extended by a lamellate extension I. Clamp supported marginally by two lateral bars *c* and *c’*, appearing as semicircular in flattened specimens. AA and AL fields with numerous tiny epidermal expansions. Anterior filed AL within a round muscular sucker.

Posterior region supported by 5 bars, *b*, *d*, *d’*, *e*, *e’*. Median bar *b*, articulated ventrally to the distal T-shaped side of *a*. Margins of posterior part of clamp supported by two short peripheral bars e and e’ and basal bars d and d’: e and e’ curved, bordering lateral portion of posterior jaw; *d* and *d’* lunar shaped, articulated at the base of *b*. In posterior region and dorsally, PA and PL with parallel rows of small rods *g* and *g’*. Rods *g* and *g’* with several concentric arcs.

No hooks observed between posterior pair of peduncles.

Oral suckers paired, rounded, smooth, aseptate and opening laterally (Fig. 2D). Pharynx ovoid, muscular. Oesophagus short. Intestinal bifurcation immediately anterior to pharynx.

Caeca long, with numerous lateral and axial diverticula, fused posteriorly to testes and extending into haptor. Testicles post-ovarian, follicular, numerous in intercaecal field of equatorial region and concealed by vitellarium. Vas deferens dorsal, sinuous, extending anteriorly along body midline to genital atrium. Genital atrium post-bifurcal, muscular, armed with 6 curved hooks (Fig. 2B).

Ovary median folded (Fig. 2F). Oviduct not observed. Oötype postovarian, surrounded by mass of Mehlis’ glands. Uterus rectilinear, running along body midline and opening into genital atrium. Seminal receptacle oval and large. Junction between seminal receptacle and the rest of the genitals not observed. Genito-intestinal canal short, originating from left intestinal branch. Vitelline follicles large, coextensive with intestinal caeca, extending from genital atrium to end of haptor; vitelline follicles nearly occupying all body proper and haptor and penetrating clamps peduncles for a short distance.

Vitelloducts Y-shaped. Dorsal transverse vitelloducts fused in middle region; common vitelline duct median, fairly long. Vagina absent. Eggs extended at each end by a short polar filament (Fig. 2C).

### Morphology of *Choricotyle* cf. *chrysophryi* Van Beneden & Hesse, 1863 (Fig. 4)

*Type-host*: *Sparus aurata* Linnaeus (jun. synonym *Chrysophrys aurata* (Linnaeus)), gilthead seabream (Sparidae Rafinesque).

**Figure 4:**
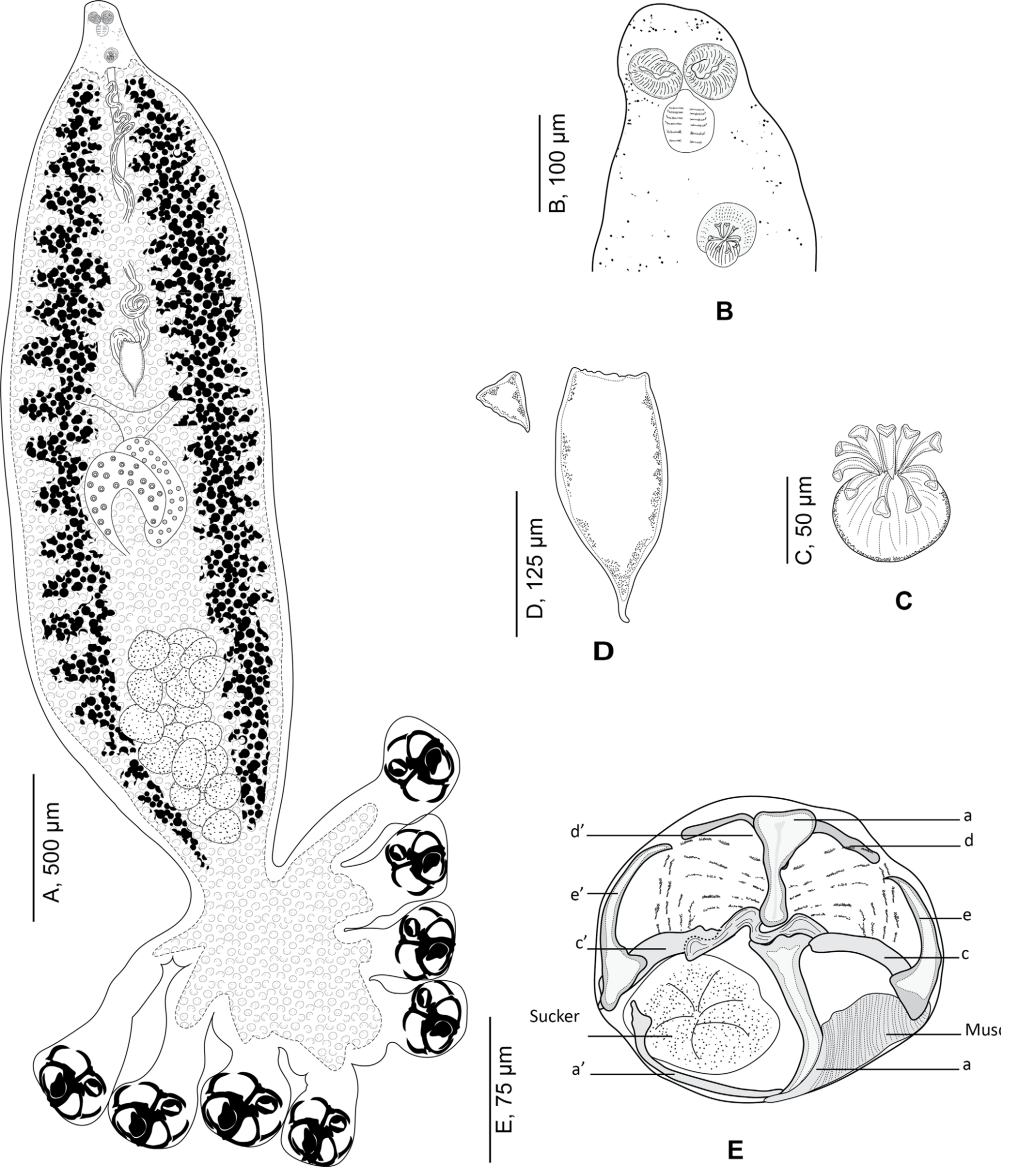
*Choricotyle* cf. *chrysophryi* Van Beneden & Hesse, 1863. A, whole body, MNHN HEL1329; B, anterior part showing relative position of prohaptoral suckers and male copulatory organ, MNHN HEL 1329. C, male copulatory organ, MNHN HEL1329; D, egg, MNHN HEL1329; E, clamp, MNHN HEL1333.

**Figure 5:**
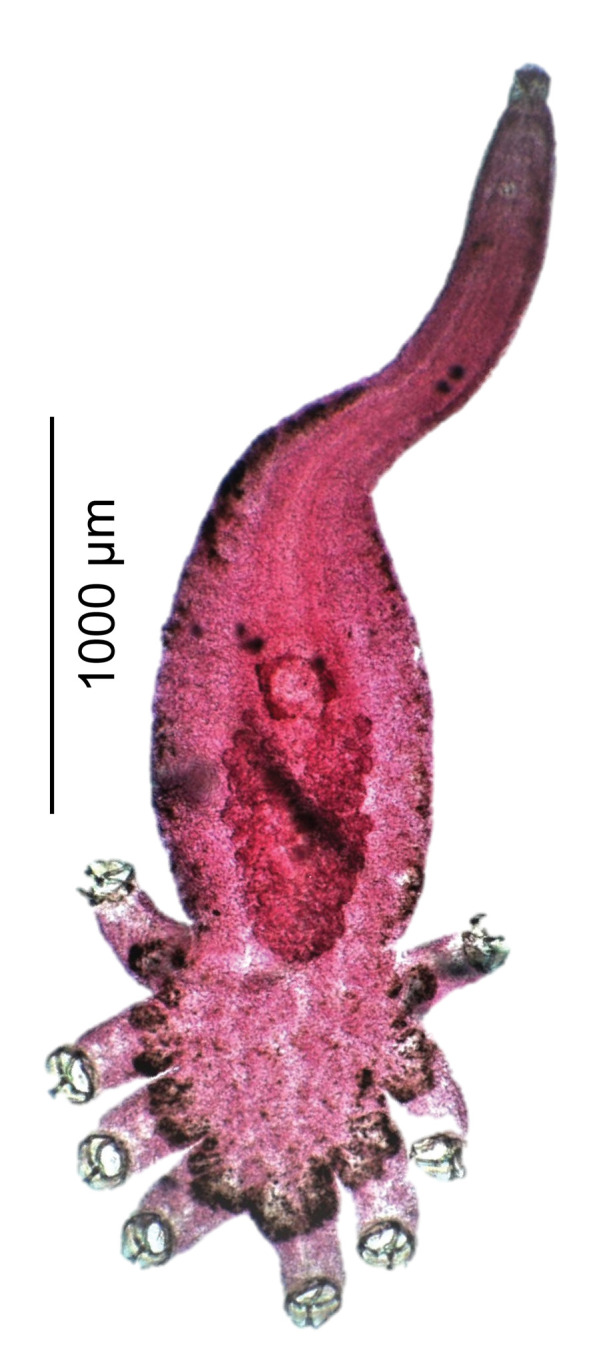
Photograph of a specimen of *Cyclocotyla bellones* on slide (MNHN HEL1317). Carmine staining (red). Note that the walls of the intestine are lined by dark black pigment from indigested blood, especially in the distal parts of the haptor.

*Additional hosts*: *Pagellus bogaraveo* (Brünnich) (jun. syn. *Pagellus centrodontus* (Delaroche)), blackspot seabream; *Spondyliosoma cantharus,* black seabream (Linnaeus); *Boops boops* (Linnaeus), bogue; *Pagellus erythrinus* (Linnaeus), common pandora; *Diplodus sargus* (Linnaeus), white seabream; *Dicentrarchus labrax* (Linnaeus), European seabass; *Pagellus acarne* (Risso), axillary seabream (this paper). See Table 4 for references

**Table 4:** Hosts and localities of *Choricotyle chrysophryi* reported in the literature.

Host/locality	Reference
*Sparus aurata* (type-host)	[94]
North-East Atlantic, off Brest, France	
*Pagellus bogaraveo*	
North–East Atlantic, off Ireland	[82]
North–East Atlantic, off Plymouth	[60]
Mediterranean, off Algeria	[51]
*Pagellus acarne*	
Mediterranean, off Algeria	[51]
Mediterranean, off Montenegro	[80]
Mediterranean, off France*	[67]
*Pagellus erythrinus*	
Mediterranean, off Montenegro	[80]
Mediterranean, off France	[92, 95]
*Diplodus sargus*	
Mediterranean, off Montenegro	[80]
Mediterranean, off Algeria	[8]
*Spondyliosoma cantharus*	
Mediterranean, off Turkey	[2]
Mediterranean, Aegean Sea	[75]
*Boops boops*	
Mediterranean, off Turkey	[2]
*Dicentrarchus labrax*	
Mediterranean, off Turkey	[3]

* Identified as *Choricotyle* cf. *chrysophryi.*

*Type-locality*: Brest, Atlantic Ocean [94].

*Additional localities*: Atlantic: Ireland, Plymouth. Mediterranean: Turkey, Montenegro, Aegean Sea, France, and off Bouharoun, Algeria (36° 37′ 24″ N, 2° 39′ 17″ E) (this study).

*Specimens from Algeria*, from gills of *Pagellus acarne*. Vouchers deposited in the collection of the Muséum National d’Histoire Naturelle, Paris (MNHN HEL1327–HEL1336). Vouchers with molecular information, 3 specimens mounted on slide, a small lateral part cut off and used for molecular analysis, deposited in the collections of the Muséum National d’Histoire Naturelle, Paris, see [15].

Based on 14 specimens. Body swollen in its posterior part and fusiform in its anterior part (Fig. 4A).

Haptor semicircular, bearing four pairs of pedunculated clamps. Peduncles short, not containing parts of intestines nor vitelline follicles; length of peduncles decreasing anteroposteriorly, fourth pairs of peduncles the smallest.

Clamps circular, typically diclidophorid in structure. Clamps with two regions, an anterior region and a posterior region. Clamps with eight sclerites: *d*, *e*, *c* on the right; *e’*, *c’*, *a’* on the left and two large hollow median sclerites *a* and *b* (Fig. 4E). Sclerite *b* I-shaped with two short anterior lobes; sclerite *a* J-shaped curved distally terminating far from the sucker’s margin; sclerites *b* and *a* articulated on each other. Ventrally, sclerite *a* bearing on its distal part a large transversal lamellate extension. Lateral sclerites *c* and *c’* slightly curved, articulated dorsally on proximal part of *a*. Lateral sclerites *d* and *d’* curved, V-shaped, situated in proximal part of the clamp and articulated ventrally to *b*. Median lateral sclerites *e* and *e’* rising dorsally to *c* and *c’*. Proximally, clamps supported dorsally by several small rods. Distally, a well-developed muscle connecting sclerites *e* and *a*.

Mouth subterminal. Oral suckers subcircular (Fig. 4B). Pharynx circular. Caeca with lateral branches, extending posteriorly; caeca not confluent posteriorly and do not enter haptor. Genital atrium mid-ventral, muscular, armed with 9 curved hooks (Fig. 4C). Testes postovarian. Vas deferens extending anteriorly. Ovary median, complex and folded. Oötype fusiform. Mehlis’ glands located in posterior part of oötype. Seminal vesicle voluminous. Oviduct short. Transverse vitelline ducts fused immediately anterior to ovary. Vitellarium not extending into haptor. Eggs fusiform (Fig. 4D) with one posterior filament.

### Morphology of the body of closely related monogeneans

**Figure 6:**
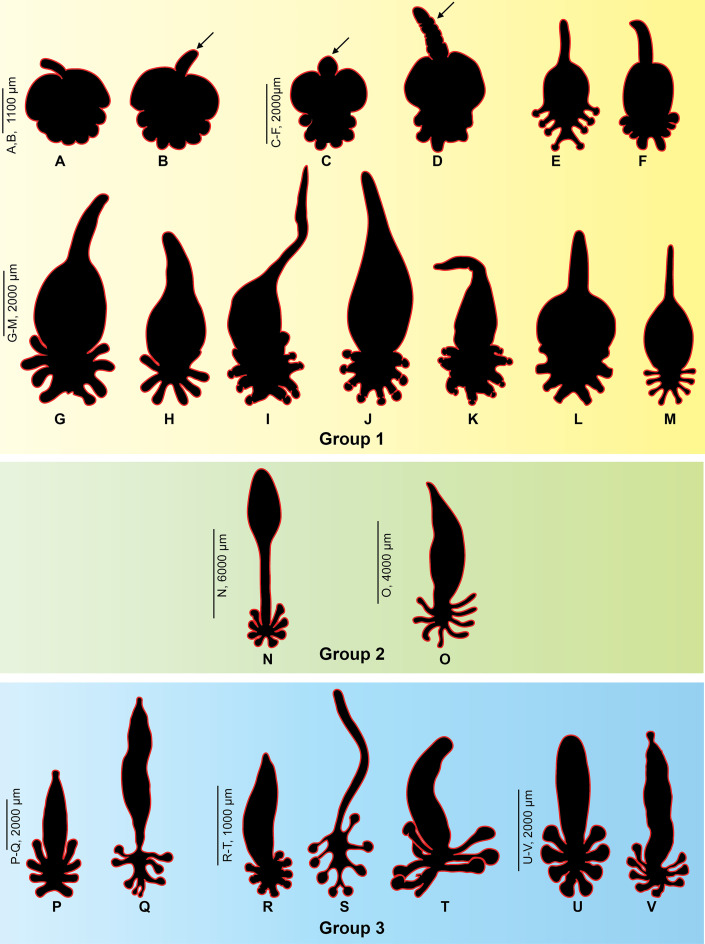
General body shapes of diclidophorid monogeneans from isopods, fish buccal cavity and fish gills. Group 1, specimens from parasitic isopods. Group 2, specimens from parasitic isopods and/or mouth. Group 3: specimens from gills. A–D, G–K, *Cyclocotyla bellones*. E, F, *Diclidophora merlangi*. L, *Allodiclidophora squillarum*. M, *Choricotyle smaris*. N, *Neoheterobothrium affine*. O, *Choricotyle elongata*. P, *Choricotyle chrysophryi*. Q, *Echinopelma neomaenis*. R, *Choricotyle hysteroncha*. S, *Choricotyle multaetesticulae*. T, *Hargicotyle louisianensis*. U, *Choricotyle labracis*. V, *Orbocotyle prionoti*. The arrows point to the anterior stem found only in species that are parasitic on isopods.

The silhouettes of the body of several diclidophorids from parasitic isopods are shown in Figure 6. Data are in Table 2. Our comparison includes 13 species: 4 of these species were collected from isopods parasite in the mouth of fish; one species from the buccal cavity; one from the buccal cavity and parasitic *Cymothoa*; the remaining 7 species of diclidophorids infect gills of fishes. The anterior extension, the stem, was observed only in diclidophorids which dwell on parasitic isopods (Figs. 6A–6M) but never in specimens that infect gills (Figs. 6P–6V).

**Figure 7:**
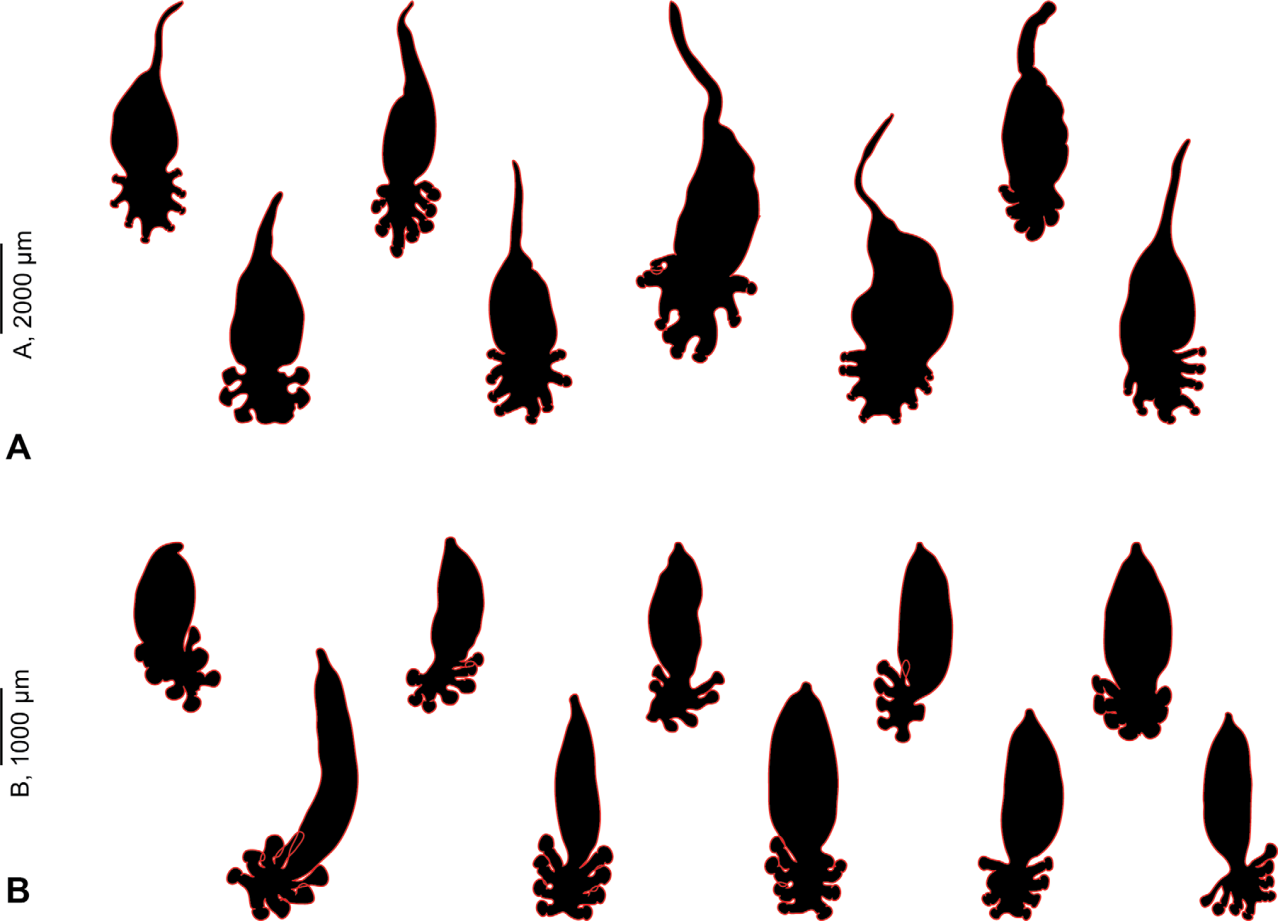
General body shapes of two closely related diclidophorids, *Cyclocotyla bellones* (A) (MNHN HEL1312, 1314-16, 1318-21) and *Choricotyle* cf. *chrysophryi* (B) (MNHN HEL1327-36).

A comparison of silhouettes of two diclidophorids collected in the present study, *Cy. bellones* and *Choricotyle* cf. *chrysophryi* shows that the stem was found only in the former species (Fig. 7).

### Intestinal content and diet of *Cyclocotyla bellones* Otto, 1823

The walls of the intestine of *Cy. Bellones* were lined by scattered dark-black pigments, especially in its distal part in the haptor; upper parts of the intestinal branches, especially near the pharynx tended to be unpigmented (Fig. 5). Based on our experience with other monogeneans, we interpret the black pigment as haematin from fish blood.

## Discussion

### Taxonomy

Hyperparasitic polyopisthocotyleans were often placed in different genera based on characters that are now considered of little generic significance, or considered synonyms with little justification (Table 5). Otto (1823) established the genus *Cyclocotyla* Otto, 1823 with its type-species *Cy. bellones* Otto, 1823, allegedly collected from the skin of the dorsal side of the garfish *Belone belone* (Linnaeus) off Naples, Italy [72]; the original description and illustrations were limited to external characters and general body shape. In a revision of the Diclidophoridae, Price (1943) revived the genus *Cyclocotyla*, taking *Cy. bellones* as type species [78, 91], and considered *Cy. bellones*, *Cy. smaris* and *Cy. squillarum* as independent species [78]. However, Palombi (1949) synonymised *Diclidophora smaris* (Ijima, 1884) Goto, 1894 with *Cy. bellones* [74]. *Cyclocotyla* was later considered a genus inquirendum by Sproston (1946) [91], and Yamaguti (1963) [100]. We do not comment any further on this taxonomic confusion because it cannot currently be resolved, since DNA sequences from monogeneans from different hosts are currently unavailable. Here, we follow Price (1943) and consider *Cy. bellones* to be a valid species, as Euzet & Trilles (1961) did [36, 78].

**Table 5:** Combinations, hosts and localities of *Cyclocotyla* spp. in the literature. The current combination is in bold.

Parasites	Synonyms	Type host	Type locality	Source
***Cyclocotyla bellones***	*Cyclobothrium charcoti*	*Belone belone*	Italy, Mediterranean Sea	[72]
*Otto, 1823*	Dollfus, 1922			[35]
*Cyclocotyla charcoti*	*Cyclobothrium charcoti* ** Dollfus, 1922	*Ceratothoa oestroides* (female), buccal cavity of *Trachurus trachurus*	Spain, Atlantic Ocean	[34, 35, 60, 100]
(Dollfus, 1922) Price, 1943 **	*Choricotyle charcoti* * (Dollfus, 1922) Llewellyn, 1941 *Cyclocotyla bellones*			
	***Allodiclidophora charcoti*** (Dollfus, 1922) Yamaguti 1963			
*Cyclocotyla elongata* (Goto, 1894) Price, 1943*	*Diclidophora elongate* Goto, 1894**	Buccal cavity of *Dentex tumifrons*, *Cymothoa* parasitic in this cavity	Japan, Pacific Ocean	[45, 60]
	***Choricotyle elongata*** (Goto, 1894) Llewellyn, 1941			
*Cyclocotyla smaris* ** (Ijima, in Goto, 1894) Price, 1943	*Diclidophora smaris* **Ijima, in Goto, 1894 *Octobothrium smaris* **Ijima, in Goto, 1894 ***Choricotyle smaris*** **(Ijima, in Goto, 1894) Llewellyn, 1941	Cymothoa of buccal cavity of *Spicara smaris*	Italy, Mediterranean Sea	[45, 60, 78]
*Cyclocotyla hysteroncha* Fujii, 1944*	***Choricotyle hysteroncha*** (Fujii, 1944) Sproston, 1946	Gills of *Haemulon striatum*	USA, Atlantic Ocean	[43, 91]
*Cyclocotyla labracis* (Cerfontaine, 1895) Price, 1943	***Choricotyle labracis*** (Cerfontaine, 1895) Llewellyn, 1941	Gills of *Dicentrarchus labrax*	North Sea, Atlantic Ocean	[19, 60, 78]
*Cyclocotyla louisianensis* * (Hargis, 1955)	***Hargicotyle louisianensis*** (Hargis, 1955) Mamaev, 1972	Gills of *Menticirrhus* americanus	USA, Atlantic Ocean	[47, 64]
*Cyclocotyla multaetesticulae* * Chauhan, 1945	***Choricotyle multaetesticulae*** (Chauhan, 1945) Sproston, 1946	Gills of a marine fish *Pellona* sp.	India, Indian Ocean	[25, 91]
Cyclocotyla neomaenis*	***Echinopelma neomaenis*** (MacCallum, 1917) Raecke, 1945	Gills of *Lutjanus analis*	USA, Atlantic Ocean	[63, 81]
(MacCallum, 1917) Price, 1943				
*Cyclocotyla prionoti* * (MacCallum, 1917) Price, 1943	***Orbocotyle prionoti*** (MacCallum, 1917) Euzet & Suriano, 1975	Gills of *Prionotus carolinus*	USA, Atlantic Ocean	[78]
	*Diclidophora neomaenis* **MacCallum, 1917 *Choricotyle neomaenis* (MacCallum,1917) Llewellyn, 1941 **			
*Cyclocotyla squillarum* **	*Mesocotyle squillarum* Parona & Perugia, 1889	Ovigerous lamellae of *Bopyrus squillarum*	Italy, Mediterranean Sea	[76]
(Parona & Perugia, 1889)	***Allodiclidophora squillarum*** (Parona & Perugia, 1889)			

* Junior synonym.

** Senior synonym.

*Cyclocotyla bellones* has been recorded on several hosts and from different localities. Nonetheless, the mention of a fish (“garfish”) as type-host of *Cy. bellones* has further obscured the status of this monogenean. Otto (1833) collected a single specimen, allegedly on the dorsal side of the host fish. We interpret this as accidental parasitism, or simply human error, and we note that *Cy. bellones* was not found on the fish itself in later studies on the parasitofauna from this host [11, 24].

### A parasite of fish or of the isopod?

A more intriguing problem is discerning the actual host of *Cy. bellones*. Is it a parasite of the crustacean or of the fish? Dollfus (1922) suggested that *Cy. bellones* was a parasite of the fish *T. trachurus* found “fortuitously” with *Cymothoa species*, rather than a parasite of this isopod [34]. Goto (1984) suggested the same for *Diclidophora smaris* collected from the mouth of a *Spicara smaris* and from the caudal segment of a *Cymothoa* [45]. Similar questions have been addressed for the monopisthocotylean *Udonella* spp. [59, 88, 89].

However, to determine the host of a parasite, we have to determine the one that assures its habitat, nutrition, and locomotion. As both the monogenean and the crustacean depend on the fish for their mobility, the later (locomotion and/or dispersal) can be neglected. We addressed the following questions:

1. Does the monogenean live on the crustacean or on the fish?
2. Does the monogenean feed on the crustacean or the fish?
3. Are there morphological adaptations in the body shape of the monogenean?

Does the monogenean live on the crustacean or on the fish?

Euzet & Trilles (1961) found *Cy. bellones* often attached to the crustacean (telson, pleon, rarely perion) and exceptionally in the buccal cavity of the fish, the palate and the internal edge of the upper lip [36]. However, Power et al. (2005) stated “*Microcotyle erythrini* and *Cy. bellones* were found attached to the gills” [77]. In this study, we found the monogenean only on the isopod, generally on its dorsal part, and never on the gills of the fish. We consider that this monogenean attaches itself to the isopod.

Euzet & Trilles (1961) found *Cy. bellones* almost exclusively on female isopods, and our results confirm this observation (Fig. 1). This might be an evolutionary adaptation for a more permanent substrate since it is known that male copepods have a shorter life span than females [88].

The answer to the first question is that *Cy. bellones* lives on the isopod, not on the fish gills.

It is noteworthy that, for the similar case of the monopisthocotylean *Udonella* sp., Carvajal et al. (2001) found no trace of the alteration of tissue in the copepod, thus excluding a parasitic relationship between the monogenean and the copepod [18, 88].

2. Does the monogenean feed on the crustacean or the fish?

Firstly, it should be noted that the isopod is protected by a strong arthropod cuticle and that nothing in the anterior part of the monogenean (mouth and prohaptor) suggests specialised morphological adaptation to pierce this cuticle, since this part is similar to most polyopisthocotylean monogeneans.

Secondly, the walls of the oesophagus and of the intestine of *Cy. bellones* are lined by dark black pigment resembling those observed in most polyopisthocotyleans [52]. These pigments are derived from ingested host blood, which suggests that *Cy. bellones* takes its nutrients from the fish. It is well known that polyopisthocotyleans cumulate blood meals and egest them at intervals via the mouth; the indigestible haematin thus appears as black pigment [52].

Hence, it is more likely that *Cy. bellones* uses isopods merely as an attachment substrate whilst feeding on blood fish.

The same was suggested for the monopisthocotylean capsalid *Capsala biparasitica* (Goto, 1894), regarded by Goto as neither commensal nor parasite (it lives on an isopod and attaches its egg to it but feeds on the fish) [45]. The udonellid *Udonella* spp. is also known for feeding directly on the mucus of the fish host [18, 40, 46, 71, 88] supplemented occasionally by epidermal cells, but not on the caligids to which they attach [39]. Sproston (1946) considered *Udonella* spp. as “feeding on mucus and gill epithelium of the fish, ‘kicked’ back by the copepod” [91]. None of these previous studies suggested that the monogeneans were feeding on the copepod tissues or body fluids.

The answer to the second question is that *Cy. bellones* feeds on fish blood, not on the isopod fluids.

Are there morphological adaptations in the body shape of the monogeneans?

After answering the two first questions, if *Cy. bellones* is attached on the isopod and feeds on the fish host, how does it reach the surface of the fish with its mouth?

The anterior stem is one of the most informative features in distinguishing *Cy. bellones* [72, 78]. Our results show that this it is observed only in diclidophorids that are dwelling on parasitic isopods or are in the buccal cavity and never in those infecting gills. It is likely that this stem allows the monogenean to stretch to reach the surface of the fish and feed on the fish blood; we do not know whether the monogenean stretches enough to reach the gills, or simply feeds on blood oozing from the fish mouth surface where it was damaged by the claws or buccal pieces of the isopod. It is noteworthy that parasitic isopods produce, within their saliva, immunomodulatory substances that modify the host immunological responses [88]. Also, substances with antithrombin activity against fish blood were identified in the salivary glands of adult *Ceratothoa oestroides* [86].

Finally, the attachment of *Cy. bellones* to the isopod might also be an adaptation of the monogenean to avoid the immune response from the fish host at the level of its posterior haptor, as previously suggested for udonellids [88].

Due to the high proportion of individual fish with a single specimen of monogenean, Euzet & Trilles (1961) suggested an adaptation of *Cy. bellones* for self-fertilisation, made possible by the anterior part lengthening then folding down [36]. However, no proof currently exists for self-fertilisation in this species. We do not reject this hypothesis of possible self-fertilisation [36], but we tend to see it as a “by-product” of the adaptation of body shape for feeding purposes.

The answer to the third question is that the elongated anterior stem is a morphological adaptation of the monogenean to feed on the blood of the fish, not on the isopod itself.

Finally, we note that the *absence* of any adaptation in the anterior part of the monogenean and its mouth is also a major argument against the monogenean feeding on the crustacean. Clearly, feeding on an arthropod and piercing its strong cuticle would require major changes in this part of the body. A parallel case is found in land planarians (Tricladida, Geoplanidae), which have species feeding on either soft body preys or on arthropods: considerable differences in anatomy are found between the two groups [10]. Nothing similar was found in monogeneans dwelling on crustaceans, especially in *Cy. bellones* studied here.

A limitation of our study is that we did not investigate the monogenean for physiological adaptation for feeding on the haemolymph of a crustacean. This could be done by demonstrating the presence of newly acquired enzymes, and/or the absence of enzymes related to the digestion of vertebrate haemoglobin. Techniques required would involve a study of expressed proteins and/or their genes, including a comparison with a close blood-feeding relative. We note here that such a new physiological novelty would require significant modifications of the proteome and genome of the monogenean.

## Conclusion

The arguments presented above suggest that *Cy. bellones* shows morphological adaptation to feed on the fish, not the isopod. We consider that the anterior stem of the body of *Cy. bellones* is an anatomical adaptation for nutrition on the host fish, and we found no evidence of specialised adaptation for perforating the isopod cuticle. We conclude that *Cy. bellones* feeds on blood from the fish and thus is not a parasite of the crustacean.

If the monogenean is not a parasite of the crustacean, then what is it? The association between *Cy. bellones* and the isopod is epibiosis (see Definitions in Table 6); the monogenean is an epibiont and the parasitic crustacean is its basibiont. Epibiosis is not a term widely used by parasitologists: it appears only once in the multi-authored 565-page volume on Marine Parasitology edited by Rohde [85] and is nowhere to be found in Mehlhorn’s 1592-page Encyclopedia of Parasitology [65]. It has only two occurrences in Levin’s 4529-page Encyclopaedia of Biodiversity [56] in a chapter on the origin of Eukaryotes, unrelated to the subject of our study.

**Table 6:** Various definitions of epibiosis in the literature.

Definition	Reference
Any relationship between two organisms in which one grows on the other but is not parasitic on it.	[26]
A relationship between two organisms, one of which lives or grows on the other, but is not parasitic on it.	[99]
The spatial association between a substrate organism (“basibiont”) and a sessile organism (“epibiont”) attached to the basibiont’s outer surface without trophically depending on it.	[97]
A spatially close association between 2 or more organisms belonging to the same or different species.	[98]
Epibiosis is a facultative association of two organisms: the epibiont and the basibiont. The term “epibiont” includes organisms that, during the sessile phase of their life cycle, are attached to the surface of a living substratum, while the basibiont lodges and constitutes a support for the epibiont.	[37]

We suggest that *Cy. bellones* should be considered an epibiont of the parasitic crustacean, as it uses it merely as an attachment substrate while feeding on fish blood. *Cyclocotyla bellones* is thus both an epibiont on the crustacean and a parasite of the fish. It could be considered a hyperparasite only in terms of location (it dwells on a parasite), but not in terms of nutrition (it does not feed on a parasite but on a host which is not a parasite).
